# P-61. Perceived Importance of Adult Immunizations among HIV Providers in New Orleans, Louisiana

**DOI:** 10.1093/ofid/ofae631.268

**Published:** 2025-01-29

**Authors:** Tat Yau, Blane Edwards, Lauren Richey

**Affiliations:** LSU Health New Orleans School of Medicine, New Orleans, Louisiana; LSU Health New Orleans, New Orleans, Louisiana; LSU Health Sciences Center New Orleans, New Orleans, Louisiana

## Abstract

**Background:**

Immunizations are key to primary care for people with HIV (PWH). Unfortunately, immunization rates among PWH are reportedly low. In addition to patients’ preferences, limited time and acute issues, knowledge gap and beliefs about vaccine among clinicians could negatively impact their recommendations, which would ultimately affect vaccine uptake for PWH.

Table 1

**Figure:**
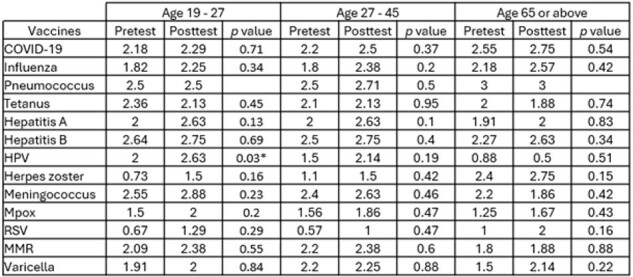

Survey results about perceived importance of vaccines of interest for people with HIV in different age groups prior to (pre-test) and after (post-test) education session

**Methods:**

This was a clinical quality improvement project at HIV Outpatient Program (HOP) at University Medical Center, New Orleans in 2023. A pretest survey was conducted to evaluate providers’ perceived importance of the vaccines of interest for PWH in different age groups: aged 19 to 26, 27 to 45, and 65 or above. Participants were asked to rank the vaccines on a scale of 0 to 3: 0 means the vaccine is not indicated; 1 means the vaccine is optional; 2 means the vaccine is important; and 3 means the vaccine is very important. The pre-test was followed by an educational session to review updates of adult immunizations tailored to PWH. Following this a post-test survey was collected to assess changes in perceived importance among participants.

Image 1

**Figure:**
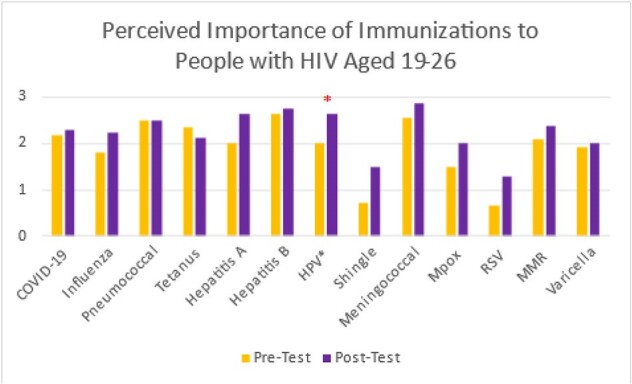

Perceived importance of immunizations among PWH aged 19 to 26

**Results:**

12 physicians participated in this study. Participants’ perceived importance of most immunizations improved after education (table 1 and figure 1-3). The perceived most important vaccines after education, defined by a score of 2.5 or above, were: meningococcus, hepatitis B, hepatitis A, HPV, and pneumococcus vaccines among PWH aged 19 to 26; hepatitis B, pneumococcus, hepatitis A, meningococcus, and COVID-19 vaccines among PWH aged 27 to 45; pneumococcus, COVID-19, herpes zoster, hepatitis B, and influenza vaccines among PWH aged 65 or above. There was a statistically significant increase in the perceived importance of HPV vaccines among PWH aged 19 to 26 after education. In addition, all participants thought pneumococcus vaccine is very important (score of 3 in both pre and posttests) for PWH aged 65 or above.

Image 2

**Figure:**
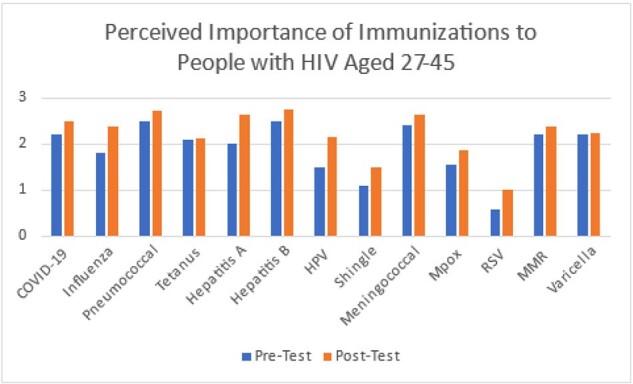

Perceived importance of immunizations among PWH aged 27 to 45

**Conclusion:**

Educational training among HIV providers raised awareness of the importance of immunization. After the training session, we noticed a 46% increase in total number of PWH aged 19 to 26 who completed HPV vaccine series. Similar approach could be implemented to general practitioners to improve vaccine uptake in primary care.

Image 3

**Figure:**
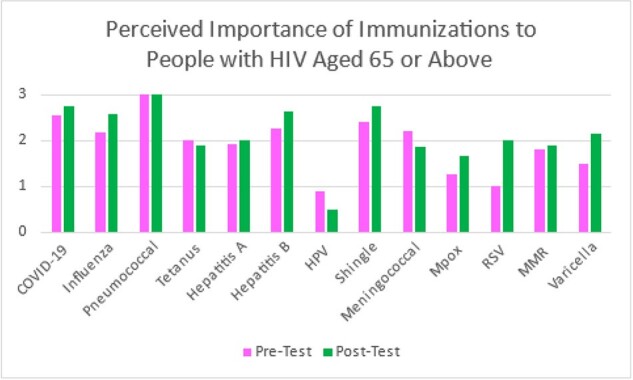

Perceived importance of immunizations among PWH aged 65 or above

**Disclosures:**

**All Authors**: No reported disclosures

